# Correlations of perioperative coagulopathy, fluid infusion and blood transfusions with survival prognosis in endovascular aortic repair for ruptured abdominal aortic aneurysm

**DOI:** 10.1186/s13017-016-0087-0

**Published:** 2016-06-17

**Authors:** Yohei Kawatani, Yoshitsugu Nakamura, Hirotsugu Kurobe, Yuji Suda, Takaki Hori

**Affiliations:** Department of Cardiovascular Surgery, Chiba-Nishi General Hospital, 107-1 Kanegasaku, Matsudo-Shi, 2702251 Chiba-Ken Japan

**Keywords:** Ruptured abdominal aortic aneurysm, Endovascular aortic repair, EVAR, Blood transfusion, Crystalloid infusion

## Abstract

**Background:**

Factors associated with survival prognosis among patients who undergo endovascular aortic repair (EVAR) for ruptured abdominal aortic aneurysms (rAAA) have not been sufficiently investigated. In the present study, we examined correlations between perioperative coagulopathy and 24-h and 30-day postoperative survival. Relationships between coagulopathy and the content of blood transfusions, volumes of crystalloid infusion and survival.

**Methods:**

This was a retrospective study of the medical records of all patients who underwent EVAR for rAAA at Chiba-Nishi General Hospital during the period from October 2013 to December 2015. Major coagulopathy was defined using the international normalized ratio or activated partial thromboplastin time (APTT) ratio of at least 1.5, or platelet count less than 50 × 10/l. We quantified the amounts of blood transfusions and crystalloid infusions administered from arrival to the hospital to admission to ICU following operations.

**Results:**

Coagulopathy among patients with rAAA was found to progress even after they had presented at the hospital. No statistically significant correlation between preoperative coagulopathy and mortality was found, although a significantly greater degree of postoperative coagulopathy was seen among patients who died both within 24-h and 30 days postoperatively. Among patients with postoperative coagulopathy, lesser quantities of fresh frozen plasma (FFP) compared with red cell concentrate (RCC) were used during the period from hospital arrival to postoperative ICU entry. In both groups of patients who did not survive after 24-h and 30 days, FFP was used less than RCC. Large transfusions of crystalloids administered during the periods from hospital arrival to surgery and from hospital arrival to the end of surgery were associated with postoperative incidence of major coagulopathy, death within 24-h, and death within 30 days.

**Conclusion:**

Coagulopathy progressed during care in the emergency outpatient clinic and operations. Postoperative coagulopathy was associated with poorer outcomes. Smaller FFP/RCC ratios and larger volumes of crystalloid infusion were associated with development of coagulopathy and poorer prognosis of survival.

**Trial registration:**

This study is retrospectively registered in UMIN Clinical Trials Registry (Registration 19 April 2016, registered number is R000025334 UMIN000021978).

## Background

Ruptured abdominal aortic aneurysm (rAAA) is a fatal condition, with mortality rates of 38 to 50 % reported, even in cases where surgery is performed [1–3]. Open repair has generally been the standard operation; however, recent reports have indicated that endovascular aortic repair (EVAR) is equally effective [4]. In a randomized controlled trial, EVAR was found to be not inferior to open repair for treating rAAA [5].

Several studies have found associations between treatment success in open repair of rAAA and factors including advanced age, female sex [6], preoperative kidney failure, chronic obstructive pulmonary disease history [7], and deranged clotting [8]. But, few studies have investigated EVAR for rAAA, and we found limited data examining correlations between perioperative coagulopathy and survival. Furthermore, no data are available concerning preoperative treatment strategies, such as fluid and blood transfusions.

Coagulopathy is thought to be closely linked with rAAA survival [9]. Additionally, an association has been reported between coagulopathy and incidence of abdominal compartment syndrome, a complication of EVAR for rAAA [10]. Therefore, coagulopathy may also be an important factor determining survival prognosis after EVAR.

Similar to rAAA, trauma–induced coagulopathy (TIC) can be detrimental to a patient’s overall condition from hemorrhagic shock and is known to worsen prognoses. Moreover, it is known that high doses of crystalloids can further worsen prognoses by contributing to TIC [11]. Early use of blood products is thought to improve prognoses [12]. Among patients undergoing EVAR for rAAA, we expected to find a correlation between coagulopathy incidence and postoperative survival prognosis, the volume and content of blood transfusions, and survival.

We retrospectively investigated associations between preoperative and postoperative coagulopathy in patients who underwent EVAR for rAAA at our hospital and 24-h and 30-day postoperative survival. Additionally, we examined how crystalloid infusion and blood transfusion correlated with coagulopathy incidence. We also examined the relation of crystalloid infusion and blood transfusion with 24-h and 30-day postoperative survival.

## Methods

### Patient selection

Subjects were all patients who underwent EVAR for rAAA at our hospital during the period from October 2013 to December 2015. Diagnosis of rAAA was made using simple computed tomography (CT) or contrast-enhanced CT. Observation of hematoma led to a diagnosis of rAAA. Patients who underwent emergency surgery for symptomatic abdominal aortic aneurysms were excluded where imaging did not show evidence of rupture.

This was a retrospective observational study. Prior to use of treatment data, consent was obtained in all cases from patients themselves or proxies with permission to make decisions on behalf of patients. The study was approved by a local ethical committee (approval No. TGE 00576-025).

### Data collection

Information was gathered from medical, nursing, medication, and emergency medical service records. The results of blood tests performed when patients were admitted to the emergency room were used as the preoperative values, and results from tests performed when patients were admitted to the ICU after surgery were used as the postoperative values. Major coagulopathy was defined by an activated partial thrombin time ratio (APTT) greater than 1.5, by a prothrombin time and international normalized ratio (PT-INR) greater than 1.5, or by a platelet count less than 50 × 10/l. To investigate the content of blood products used, we calculated RCC/FFP ratios and differences (RCC-FFP) based on the volumes of blood products used during the period from hospital arrival to ICU entry just after surgery.

Three treatment outcomes were observed: intraoperative death, 24-h postoperative survival, or 30-day postoperative survival.

### Pre- and intraoperative management

Decisions to administer fluid and blood transfusions, and about which compositions should be used, were made by the physicians in charge of outpatient care from hospital arrival to the start of surgery. In the period after patients entered the operating room, during surgery, and until the patients arrived in the ICU these decisions were made by anesthesiologists.

In principle, surgeries commenced under local anesthesia and general anesthesia was introduced after preparation for use of an aortic occlusion balloon. General anesthesia was administered before any incisions were made if the anesthesiologist and surgeon agreed this could be done safely.

Surgeries began without the use of heparin; however, heparin was administered in cases where it was possible to insert a balloon to occlude the aorta in order to ensure that activated clotting time was at least 200 s. Protamine was used to reverse the effects of heparin at the end of the operation, administered at a dose equivalent quantity. Following the end of the operation, all patients were transferred to the ICU while sedated and connected to an artificial respiratory device.

### Statistical analysis

For continuous variables, means and standard deviations were calculated. Categorical variables were presented as *n* (percentage of total [%]). The Mann-Whitney test was used for analysis of continuous variables, whilst categorical variables were compared using the chi-square test. Differences were considered statistically significant at a *p*-value of <0.05.

All statistical analyses were performed on a personal computer using the statistical software package SPSS for Mac (Version 22; SPSS Inc., Chicago, IL, USA).

## Results

### Treatment outcome

Forty-seven patients presenting with rAAA in our hospital were enrolled in the study. One patient was considered unfit for surgery. Of the 46 patients who went to theater, 25 underwent EVAR and thus were included in our analyses.

With regard to treatment outcomes, no patients died in the operating theater, whilst three died due to abdominal compartment syndrome (ACS) within 24-h postsurgery, and an additional two died between 24-h and 30 days postsurgery. One of these two patients died due to respiratory failure and one of cancer.

### Comparison of preoperative and postoperative examination values

Data from the 25 subjects before and after surgery were compared using paired t-tests. Statistically significant changes were seen in APTT and PT-INR, which increased, and platelet counts, which decreased. No patients presented with major coagulopathy prior to surgery, although nine patients experienced major coagulopathy afterward (Table 1).

**Table 1:** Comparison of the preoperative and postoperative coagulation profiles in all participants

	Preoperative	Postoperative	*p*
APTT (second)	27.8 +/- 5.2	47.3 +/- 30.1	0.002
PT-INR	1.2 +/- 0.2	1.4 +/- 0.2	<0.001
PLT counts (10^4^/μL)	16.3 +/- 5.2	9.9 +/- 4.7	<0.001
Major coagulopathy (*n*)	0	9	<0.001

*APTT* activated partial thromboplastin time ratio, *PT-INR* prothrombin time and international normalized ratio, *PLT* platelet

### Coagulopathy, fluid infusion, and blood transfusion

Patients who presented major coagulopathy after surgery received larger total fluid transfusions from hospital arrival to the end of surgery. More patients in the non-survival groups for both 24-h and 30-day survival received fluid transfusions of 1.5 L (Table 2).

**Table 2:** Survival and coagulation profiles

	Survival	Non-survival	*p*
24-h survival			
*n*	22	3	
Preoperative APTT (second)	27.0 +/- 4.3	33.6 +/- 8.4	0.21
Postoperative APTT (second)	38.9 +/- 8.7	108.7 +/- 63.4	0.006
APTT change (second)	11.9 +/- 9.2	75.0 +/- 58.9	0.006
Preoperative PT-INR	1.2 +/- 0.16	1.2 +/- 0.2	0.802
Postoperative PT-INR	1.3 +/- 0.20	1.5 +/- 0.28	0.295
PT-INR change	0.16 +/- 0.17	0.33 +/- 0.33	0.503
Preoperative platelet counts (10^4^/μL)	16.1 +/- 5.4	17.3 +/- 3.0	0.616
Postoperative platelet counts (10^4^/μL)	10.2 +/- 5.0	7.7 +/- 1.9	0.558
Platelet count change (10^4^/μL)	5.9 +/- 6.2	9.5 +/- 5.2	0.452
Preoperative major coagulopathy (*n*)	0 (0 %)	0 (0 %)	NS
Postoperative major coagulopathy (*n*)	6 (27 %)	3 (100 %)	0.037
NS: not significant			
30-day survival			
*n*	20	5	
Preoperative APTT (second)	26.8 +/- 4.3	32 +/- 7.0	0.119
Postoperative APTT (second)	38.1 +/- 7.9	95.7 +/- 57.9	0.002
APTT change (second)	11.3 +/- 8.9	62.7 +/- 54.1	0.002
Preoperative PT-INR	1.2 +/- 0.16	1.23 +/- 0.19	0.0.767
Postoperative PT-INR	1.4 +/- 0.2	1.5 +/- 0.2	0.148
PT-INR change	0.16 +/- 0.18	0.30 +/- 0.28	0.436
Preoperative platelet counts (10^4^/μL)	16.2 +/-5.54	16.8 +/- 2.7	0.767
Postoperative platelet counts (10^4^/μL)	10.4 +/- 5.0	7.2 +/- 1.9	0.299
Platelet count change (10^4^/μL)	−5.7 +/-6.3	−9.6 +/- 4.0	0.335
Preoperative major coagulopathy (*n*)	0 (0 %)	0 (0 %)	NS
Postoperative major coagulopathy (*n*)	5 (25 %)	4 (80 %)	0.01

*APTT* activated partial thromboplastin time ratio, *PT-INR* prothrombin time and international normalized ratio, *PLT* platelet

The values for RCC/FFP ratio and RCC-FFP were significantly larger in the group that exhibited major coagulopathy than in the group that did not.

### Coagulopathy and survival

Postoperative APTT was significantly longer in the 24-h and 30-day non-survival groups in comparison with the respective survival groups (Table 3).

**Table 3:** Postoperative major coagulopathy and blood and fluid transfusions

	Postoperative major coagulopathy		
	Non-presented	Presented	*p*
RCC (units)	5.63 +/- 5.3	13.6 +/- 7.0	0.004
FFP (units)	5.75 +/- 6.4	6.2 +/- 7.5	0.743
PC (units)	5.6 +/- 11.5	4.4 +/- 8.8	0.57
RCC/FFP ratio	1.1 +/- 0.23	2.00 +/- 0.73	0.015
RCC - FFP (units)	2.3 +/- 3.5	7.9 +/- 4.3	0.001
Preoperative crystalloid volume (mL)	750 +/- 408	1589 +/- 1071	0.022
Total crystalloid volume (mL)	2214 +/- 893	3572 +/- 1391	0.014
Total crystalloids >1.5 L (*n*)	4 (25 %)	5 (56 %)	0.137

*RCC* red cell concentrate, *FFP* fresh frozen plasma, *PC* platelet concentrate

A significantly greater number of patients in the 24-h non-survival group exhibited major coagulopathy postoperatively compared with the survival group. Similarly, there more instances of major coagulopathy were observed in the 30-day non-survival group than in the survival group.

### Fluid and blood transfusions and survival

Values for RCC/FFP ratios and RCC-FFP were significantly larger in both the 24-h and 30-day non-survival groups compared with the respective survival groups (Table 4). Patients with RCC/FFP ratios greater than 1.5 had higher risks of mortality.

**Table 4:** Survival and blood and fluid transfusions

	Survival	Non-survival	*p*
24-h survival			
RCC (units)	7.3 +/- 5.3	17.7 +/- 7.8	0.035
FFP (units)	3.6 +/- 4.2	12.0 +/- 6.9	0.02
PC (units)	5.7 +/- 12.9	13.3 +/- 11.5	0.271
RCC/FFP ratio	1.3 +/- 0.6	1.6 +/- 0.8	0.014
RCC/FFP >1.5 L (*n*)	2 (9 %)	3 (100 %)	0.018
RCC - FFP (units)	4.5 +/- 4.7	5.7 +/- 5.7	0.006
Preoperative crystalloid volume (mL)	877 +/- 615	2333 +/- 1041	0.01
Total crystalloid volume (mL)	2386 +/- 912	5083 +/-803	0.028
Total crystalloid >1.5 L (*n*)	5 (23 %)	3 (100 %)	0.037
30-day survival			
RCC (units)	6.9 +/- 5.6	15.8 +/- 7.4	0.017
FFP (units)	3.4 +/- 4.2	10.0 +/- 6.9	0.116
PC (units)	5.7 +/- 12.9	10.0 +/- 11.5	0.96
RCC/FFP ratio	1.1 +/- 0.6	1.8 +/- 0.8	0.001
RCC/FFP > 1.5 L (*n*)	1 (5 %)	4 (70 %)	0.03
RCC - FFP (units)	3.5+/-4.7	5.8+/-4.6	0.03
Preoperative crystalloid volume (mL)	847 +/- 615	2125 +/- 946	0.003
Total crystalloid volume (mL)	2341 +/- 9.6	4550 +/- 1252	0.012
Crystalloids >1.5 L (*n*)	5 (25 %)	4 (80 %)	0.01

*RCC* red cell concentrate, *FFP* fresh frozen plasma, *PC* platelet concentrate

Larger quantities of crystalloids were used in the both the 24-h and 30-day non-survival groups compared with the respective survival groups.

## Discussion

For all subjects, values for postoperative APTT and PT-INR were significantly longer than preoperative values. While there were no instances of major coagulopathy preoperatively, the condition was observed in nine patients postoperatively.

At both 24-h and 30 days postoperation, there were no significant differences in preoperative APTT, PT-INR, or major coagulopathy between the survival groups and non-survival groups. These results concur with findings from a previous study that examined factors related to survival after open repair for rAAA and found no correlation between preoperative coagulopathy and mortality [13].

Conversely, our findings show that postoperative APTT was significantly longer in the non-survival groups than in the survival groups at both 24 h and 30 days. Furthermore, APTT increased by a significantly larger proportion between pre- and postsurgery in the non-survival groups at both 24 h and 30 days.

There were more instances of postoperative major coagulopathy in the non-survival groups than in the survival groups at both 24 h and 30 days. In a previous study of patients who underwent open repair for rAAA, prolonged PT and prolonged APTT at postoperative ICU entry were associated with poorer prognoses [14]. The same appears to be true for patients treated with EVAR.

In the present study, a tendency for coagulopathy to progress during the period from hospital arrival to the end of surgery was observed among all cases, with patients who exhibited greater progression also exhibiting higher mortality risks. Efforts to control the progression of coagulopathy after hospital arrival could help to improve survival prognoses.

ACS is a serious condition that can occur after EVAR for rAAA and negatively affects survival [15]. Its mortality rate is nearly 100 % without treatment and is still high (30–60 %) with appropriate treatment [16]. ACS is thought to be caused by massive retroperitoneal hematoma and diffuse visceral edema and is diagnosed when the abdominal pressure exceeds 20 mmHg in combination with endo-organ dysfunction. Early recognition and surgical decompression of ACS is essential [17]. Coagulopathy is reportedly a risk factor for ACS [12]. In fact, all the patients who died within 24 h in the present study experienced ACS and exhibited postoperative major coagulopathy. Routine measurement of intraabdominal pressure is useful for detecting ACS. Especially in patients with coagulopathy after EVAR for rAAA, intraabdominal pressure should be monitored carefully. Additionally, we believe that maintaining clotting function by FFP blood trans fusion and limitation of crystalloid use can prevent ACS development.

In a report on trauma treatment by Veena et al. [18], greater administered quantities of crystalloids were associated with coagulopathy progression, cellular distension, heart complications, and elevated abdominal compartment pressure. In addition, incidence of ACS has been linked to large fluid transfusions [19].

In the present study, quantities of crystalloids administered were significantly higher among patients with major coagulopathy. Furthermore, quantities of crystalloids used were significantly higher in the non-survival groups at both 24-h and 30 days. Crystalloid transfusion is associated with coagulopathy onset in rAAA patients who undergo EVAR and is thought to have a negative effect on prognosis. In a study of trauma patients, cases that received large quantities of crystalloids in initial transfusions reportedly required larger blood transfusions [20]. It is possible that limiting use of crystalloids can prevent postoperative coagulopathy and can make better the prognosis of survival.

In the present study, values for both RCC/FFP ratio and RCC-FFP were larger among patients with postoperative major coagulopathy. Previously, a prospective study of patients who underwent open surgery for rAAA found that aggressive FFP and PLT administration significantly inhibited the prolongation of postoperative APTT [21].

In this study, the relatively lesser use of FFP compared with RCC appears to be associated with major coagulopathy.

Studies investigating blood transfusion composition and survival prognosis in trauma patients have indicated that an appropriate target for RCC/FFP ratios is between 3 and 4 [22]. However, a study conducted by the military found that maintaining an RCC/FFP ratio close to 1 led to increased survival rates [23]. A retrospective investigation of RCC/FFP ratios in trauma patients reported a link between a 1:1 RCC/FFP ratio and positive prognoses [24]. Furthermore, the PROPPR randomized clinical trial, which divided trauma patients into an RCC:FFP:PC = 1:1:1 group and an RCC:FFP:PC = 2:1:1 group, found that the former group achieved hemostasis and experienced less exsanguination in 24 h, although no significant differences in mortality between the two groups were observed [25]. Most of the above results suggest that quantities of FFP equal to those of RCC can be useful for improving the survival prognoses of patients with rAAA receiving EVAR by maintaining a coagulation profile and achieving hemostasis.

Our hospital does not have a protocol for blood transfusion in rAAA patients. The physician in charge determines the content of transfusions based on test results. Mean transfusion quantities were RCC 10.0 +/- 5.70 units and FFP 4.4 +/- 3.3 units, representing a RCC/FFP ratio greater than 1. On the basis of results from the present and previous studies, it appears that basing decisions for how much FFP to administer on test results may not keep up with the patient’s progression. For rAAA patients undergoing EVAR who require blood transfusions, coagulopathy may be controlled and survival prognosis improved by following a transfusion protocol that uses a 1:1 RCC/FFP ratio and administers FFP early.

## Conclusion

Coagulopathy progressed during care in the emergency outpatient clinic and the operations, and postoperative coagulopathy was associated with poorer outcomes. We suggest that smaller FFP/RCC ratios and larger volumes of crystalloid infusions potentially contributed to coagulopathy and ACS development, and poor survival prognosis. Limitations of this study include the small sample number and its retrospective design, and further investigation is needed.

## Abbreviations

ACS, abdominal compartment syndrome; APTT, activated partial thrombin time ratio; EVAR, endovascular aortic repair; FFP, fresh frozen plasma; ICU, intensive care unit; PC, platelet concentrate; PT-INR, prothrombin time international normalized ratio; RCC, red cell concentrated.
